# Tricuspid valve repair and replacement for infective endocarditis

**DOI:** 10.1007/s12055-023-01650-0

**Published:** 2023-12-16

**Authors:** Antonella Galeone, Jacopo Gardellini, Fabiola Perrone, Alessandra Francica, Gina Mazzeo, Marcello Raimondi Lucchetti, Francesco Onorati, Giovanni Battista Luciani

**Affiliations:** Department of Surgery, Dentistry, Pediatrics and Gynecology, Division of Cardiac Surgery, University of Verona, Verona, Italy

**Keywords:** Infective endocarditis, Tricuspid valve, Tricuspid valve repair, Tricuspid valve replacement

## Abstract

Infective endocarditis represents a challenging and life-threatening clinical condition affecting native and prosthetic heart valves, endocardium, and implanted cardiac devices. Right-sided infective endocarditis account for approximately 5–10% of all infective endocarditis and are often associated with intravenous drug use, intracardiac devices, central venous catheters, and congenital heart disease. The tricuspid valve is involved in 90% of right-side infective endocarditis. The primary treatment of tricuspid valve infective endocarditis is based on long-term intravenous antibiotics. When surgery is required, different interventions have been proposed, ranging from valvectomy to various types of valve repair to complete replacement of the valve. Percutaneous removal of vegetations using the AngioVac system has also been proposed in these patients. The aim of this narrative review is to provide an overview of the current surgical options and to discuss the results of the different surgical strategies in patients with tricuspid valve infective endocarditis.

**Supplementary Information:**

The online version contains supplementary material available at 10.1007/s12055-023-01650-0.

## Introduction

Infective endocarditis (IE) represents a challenging and life-threatening clinical condition affecting native and prosthetic heart valves, endocardium, and implanted cardiac devices with a mortality up to 30% in the first year [1]. Both incidence and mortality of IE have constantly increased in the last three decades despite advances in diagnostic approaches and therapeutic management [2].

Right-sided infected infective endocarditis (RSIE) account for approximately 5–10% of all cases of IE and are often associated with intravenous drug use (IVDU), intracardiac devices, central venous catheters, and congenital heart disease [3]. *Staphylococcus aureus* is responsible for 60–90% of RSIE; other causative microorganisms include coagulase-negative staphylococci and streptococci [4]. *Pseudomonas aeruginosa* and other gram-negative bacteria have also been reported in RSIE. Fungal infections are very rare and account for about 3% of all RSIE; however, fungal infections are characterized by a high mortality of up to 54% in some series [5].

The tricuspid valve (TV) is involved in 90% of RSIE cases [3]. The primary treatment of tricuspid valve infective endocarditis is based on the administration of intravenous antibiotics for 4–6 weeks. Surgery is required in 5–40% of patients [6, 7], and operative mortality rates have been reported between 5 and 15% [7–9]. Concomitant left-sided IE carries a worse prognosis than right-sided infection alone, due predominantly to its greater likelihood for invasion and abscess formation [7]. The main indications for surgery are (I) right ventricular dysfunction secondary to acute severe TV regurgitation non-responsive to diuretics, (II) respiratory insufficiency requiring ventilatory support after recurrent pulmonary emboli, (III) large residual TV vegetations (> 20 mm) after recurrent septic pulmonary embolism, (IV) simultaneous involvement of left-sided structures, and (V) persistent bacteremia after at least 1 week of appropriate antibiotic therapy [10].

The primary objective of surgery for TVIE is the radical debridement of all infected vegetations and foreign material in order to eliminate the cause of persistent sepsis and source of septic emboli in the lung [11]. Numerous interventions have been proposed for the surgical treatment of patients with TVIE, ranging from valvectomy to various types of valve repair to complete replacement of the valve [6]. However, optimal surgical treatment of TVIE still remains controversial especially in patients with IVDU given the high rates of recidivism and the inherent social concerns. Tricuspid valve repair should be attempted whenever possible, and the repair and preservation of the patient’s own valve are considered the first choice in patients with TVIE [11]. Tricuspid valvectomy, that is the removal of the TV leaflets and chordae tendinae without replacement, has been also suggested [12]. However, in patients with increased pulmonary pressure and resistance, excising the valve and leaving severe regurgitation are not advisable. When TV repair is not feasible and valve replacement is required, most surgeons choose a biological prosthetic valve [11]. Recently, percutaneous removal of vegetations using the AngioVac system has also been proposed in these patients [13, 14].

The aim of this narrative review is to provide an overview of the current surgical options and to discuss the results of the different surgical strategies in patients with TVIE.

### Tricuspid valvectomy

Tricuspid valvectomy has been proposed for TVIE in patients with IVDU given the inherent social concerns, the high recidivism rates, and the consequent risk of reoperation for prosthetic valve infection. Excision has the advantage that it can be performed quickly in the beating heart in patients with severe sepsis who could not be able to tolerate prolonged periods of cardiopulmonary bypass. Other advantages of valvectomy include the limitation of foreign material, avoidance of the need for anticoagulation therapy, and lower risk of heart block requiring a permanent pacemaker [15]. However, valvectomy without replacement results in massive tricuspid regurgitation (TR) and ventricularization of right atrial pressures and may lead to right-sided heart failure requiring TV replacement [16]. For these reasons, it should be offered as a staged or a palliative procedure.

Arbulu et al. first described in 1971 the removal of the TV leaflets and chordae tendinae without replacement for the treatment of a patient with *Pseudomonas aeruginosa* infective endocarditis [12]. Later, the authors published the results of their twenty years’ experience with the tricuspid valvectomy without replacement in 53 patients affected by TVIE [16]. All patients were addicted to heroin, and half of the patients returned to their drug addiction after surgery. Operative mortality was 11%, while the overall mortality rate was approximately 30%. Six patients (11%) required prosthetic heart valve replacement 2 days to 13 years later for medically refractory right-sided heart failure. Protos et al. [17] reported on their single-center experience with the management of 63 patients with TVIE from 2012 to 2016. The comparison between TV valvectomy, repair, and replacement showed no difference in 30-day mortality; however, patients with valvectomy had significantly lower unplanned readmission rates at 1 year. The incidence of bleeding requiring reoperation, major stroke, prolonged ventilator time, intensive care unit (ICU) stay, and overall hospital length of stay was similar in all groups. Of note, 30-day mortality in patients with TV valvectomy, repair, and replacement was 4%, 0%, and 0% respectively. The authors ascribed the discrepancy in operative mortality with the previously published reports on tricuspid valvectomy to the type of organism involved, the advancement of perioperative and critical care, and more efficacious antimicrobial therapies. In this series, a low number of patients were available at 1-year follow-up and satisfied the requirements of drug abstinence, suggesting caution in proceeding with TV replacement at the initial operation in the IVDU population. The lack of difference in perioperative outcomes, coupled with high prosthetic valve infection and readmission rates seen in patients treated with valve replacement at their initial operation, prompted the authors to consider TV valvectomy as a useful bridge to staged valve replacement in this high-risk patient population. This strategy allows patients who can maintain adequate follow-up and abstinence from IV drugs to undergo a planned, elective, staged valve replacement reoperation after the initial valvectomy, which not only allows patients the opportunity to be drug-free and infection-free but also eliminates the need for an urgent reoperation for an infected prosthetic valve [17].

A recent metanalysis compared the outcomes of TV valvectomy versus replacement for the surgical treatment of isolated TVIE [18]. A total of 752 patients from 16 studies were entered in the meta-analysis, of which 14% underwent valvectomy and 86% underwent replacement with a mean follow-up of 4.2 years. The most common indication for surgical intervention was septic pulmonary embolism in the valvectomy group and persistent sepsis in the replacement group. There was no statistically significant difference between TV valvectomy and replacement in 30-day postoperative mortality (13% vs 7% respectively), post-operative right heart failure (27% vs 11%, respectively), and recurrent endocarditis (7% vs 19%, respectively). Six-month and 1-year survival were also comparable between the two groups. There was a trend towards higher rates of delayed reoperations for TV replacement in the valvectomy group compared to initial replacement (56% vs 14% respectively) for staged TV replacement. However, at 1 year, freedom from reoperation was lower in the valvectomy group compared to those who underwent replacement (46% vs 89%, respectively), but the difference was not statistically significant. This metanalysis demonstrated that total tricuspid valvectomy without replacement is an acceptable initial therapy for TVIE in patients with IVDU and can be a life-saving measure in these patients. Tricuspid valvectomy can effectively eradicate the focus of infection with good perioperative outcomes and provide a bridge to identify those patients who will self-select as candidates for staged valve replacement [18]. Wang et al. recently reported their experience in drug-addicted patients with TVIE treated with resection of infected TV leaflets, TV annular reduction, and bidirectional Glenn shunt [19]. Bidirectional Glenn shunt diverts flow from the superior vena cava to the pulmonary artery to unload the right ventricular and may provide a stable hemodynamic condition after TV resection and offer an adequate observation period for drug abstinence. In the long-term follow-up, prosthetic TV replacement and superior vena cava reimplantation are needed in case of severe tricuspid insufficiency and worsened right ventricular function [19]. A contemporary report of the Society of Thoracic surgeons (STS) database analyzed the outcomes of isolated TV operations performed between 2011 and 2016 in 1613 patients with intravenous drug-associated TVIE and revealed that the operative mortality was significantly higher in the valvectomy group (16%) than in the TV repair (2%) and replacement groups (3%) [20]. However, compared with the repair and replacement groups, the valvectomy group had a significantly higher rate of active infection, urgent/emergency surgery, and more comorbidities and preoperative risk, such as higher white blood cell count, Mayo end-stage liver disease (MELD) score, and lower hematocrit and albumin levels. Additionally, the patients in the valvectomy group were more likely to have Medicaid or other state-supported insurance, which may be reflective of poor outcomes with poor socioeconomic status. Interestingly, cause of death analysis in the valvectomy group showed that 47% of patients died of infection, which suggests continued infection even after removal of the infected valve [20].

### Tricuspid valve repair

Successful surgical treatment of infective endocarditis requires the aggressive and extensive debridement of all vegetations and infected tissue. The majority of centers prioritize TV repair, whereby valvectomy or valve replacement is only performed after unsuccessful attempts to repair [15]. Reconstruction of the TV is certainly favored over replacement due to the high risk of reinfection, poor outcomes with valve replacement, and patient compliance issues. However, diffuse, multifocal vegetations, and extensive valve destruction often leave insufficient building materials necessary for repair after complete debridement. Thus, some authors suggest intervening as early as possible after presentation in an effort to limit tissue destruction and preserve tissue integrity to maximize the likelihood of valve repair [21]. Tricuspid valve repair techniques typically included pericardium leaflet patch repair/augmentation, bicuspidization, use of artificial chordae, and prosthetic annuloplasty [22] (see Video 1 and Video 2). The Kay bicuspidization technique represents an alternative to prosthetic ring annuloplasty [23]. Through this technique, the posterior leaflet is eliminated, and the valve contains only an anterior and septal leaflet. The suture used to reshape the annulus is brought through a pledget on both sides and tied down. Another alternative to prosthetic ring annuloplasty is the De Vega suture annuloplasty [24], in which a 3–0 polypropylene is used to suture two rows in the tricuspid annulus, around the perimeter of the tricuspid valve along the same distribution as a partial annuloplasty ring (10 o’clock to 6 o’clock). A pledget is utilized on either end of the rows of suture, and the ends are tied down and cut. The clover technique has also been proposed for the conservative management of TVIE. This technique was first described by Alfieri et al. in 2003 in patients with post-traumatic TV regurgitation and consists of stitching together the middle point of the free edges of the tricuspid leaflets, by using a 5–0 polipropylene suture without pledgets and adding a semi-rigid ring, thus producing a clover-shaped valve [25]. Fayad et al. reported their experience with the clover technique in five patients with TVIE [26, 27]. All five patients survived the surgery. During follow-up, there was no recurrent TVIE and echocardiographic evaluation showed trivial or no residual regurgitation. The absence of transvalvular gradient and TV calcification and the preservation of right ventricular function indicate the reliability of this technique.

There are important differences in tricuspid valve repair techniques that deserve mention. Leaflet patch augmentation and chordal replacement may be technically complex, particularly for surgeons not accustomed to TV repair. In TVIE associated with IVDU, avoidance of any prosthetic material during valve repair has been advocated to further limit recurring endocarditis. However, the avoidance of prosthetic material should not compromise radical eradication of all infected tissue, long-term freedom of recurring endocarditis, or valvular competence. Ring annuloplasty introduces more prosthetic material than De Vega, but the latter has been associated with TV regurgitation recurrence; Kay annuloplasty or bicuspidization can be considered if the vegetation is limited to the posterior leaflet, and vegetectomy alone may be associated with recurrent IE. Implantation of an annuloplasty ring in patients undergoing TV repair prevents future tricuspid annulus dilatation and appearance/worsening of TV regurgitation. It is necessary to stabilize valve geometry to achieve a long-term competent valve, and this is especially crucial in patients with massive destruction of one or two leaflets, which necessitates extensive repair with resection of a leaflet or a commissuroplasty [28].

Musci et al. reported on their 20-year experience with surgical treatment of TVIE in 79 patients who underwent surgery between 1986 and 2006 [7]. The authors recommend that whenever possible, the TV should be repaired with homologous or autologous pericardium to avoid the implantation of prosthetic material. If the infection is well-circumscribed and localized on an otherwise normal valve, a simple vegetectomy can be performed. After vegetectomy or excision of the perforated leaflet, the defect is closed directly or with a pericardial patch. To ensure leaflet coaptation, annuloplasty should be performed and reinforced with pericardium. The correction of defects that have developed, such as fistulas or abscess cavities, should also be performed with homologous or autologous pericardial patches [7]. In this series, there was no significant difference between the survival rates of patients after tricuspid valve replacement compared to those with tricuspid valve reconstruction: the 30-day, 1-, 5-, 10- and 20-year survival rate after reconstruction was 88%, 81%, 68%, 68%, and 57%, respectively, in comparison to 87%, 83%, 62%, 20%, and 20% after replacement. However, analysis of the survival curves shows a tendency towards better survival following tricuspid valve reconstruction in comparison to the tricuspid valve replacement group in which there is a sudden drop in the survival curve between 7 and 10 years.

Dawood et al. reviewed the outcomes of 56 patients treated for TVIE between 2002 and 2012 and reported a mortality rate of 0% [29]. Successful valve repair was obtained in 57% of the patients. The approach of the authors included debridement of infected tissue, use of untreated autologous pericardium as a patch when necessary, and placement of an undersized partial annuloplasty ring. Suture annuloplasty to avoid a prosthetic ring may result in a higher recurrence of TV regurgitation and annulus dilatation and TV regurgitation. The reinfection rate in the repair group was 0% despite the high prevalence of IVDU in this population and the use of prosthetic ring, while 21% of patients with TV replacements required late reoperations for prosthetic valve endocarditis secondary to recurrent IVDU. Patients initially treated with antibiotics tended to have a higher rate of TV replacement rather than repair compared with patients who underwent early surgery, suggesting that earlier intervention may avoid ongoing destruction of leaflet tissue and increase the likelihood of TV repair.

Tarola et al. described a complex surgical technique to reconstruct the TV in patients with grossly infected TVs with more than 50% valvular destruction [30]. This technique is based on extensive autologous pericardial patch augmentation of the destroyed TV leaflets to establish leaflet coaptation, supplemented with expanded polytetrafluoroethylene (e-PTFE) neo-chordae and annular reconstruction with a prosthetic ring. Between 2008 and 2013, this technique was applied to 12 consecutive patients with TVIE affecting more than 50% of the TV. There were no in-hospital or 30-day postoperative deaths. Early operative results following surgery demonstrated a well-functioning, competent TV with minimal regurgitation, and the rate of freedom from reoperation was 100% [30].

Similarly, Hosseini et al. reported their experience in nine patients with TVIE and periannular involvement using autologous pericardium for valve reconstruction [31]. Radical debridement was performed to provide a safe ground for pericardium implantation. Untreated pericardial patches were prepared and sutured to the remaining part of the debrided annulus. Neochordae were fashioned with e-PTFE sutures attached to the free edge of the pericardial neoleaflet. In-hospital mortality was 0%; during the follow-up, two non-cardiac-related deaths occurred, but there were no cases of recurrent endocarditis or reoperation [31].

A meta-analysis including 12 retrospective observational studies with 1165 patients and a median follow-up of 3.8 years aims to compare the early and late outcomes of valve repair versus replacement [15]. The median repair proportion was 59%, and replacement was 41% among studies. The main repair strategies were vegetectomy, De Vega procedure, annuloplasty ring, bicuspidization, and leaflet patch augmentation. Of valve replacements, 83% were bioprosthetic, and 17% were mechanical prostheses. There were no differences in perioperative mortality and long-term all-cause mortality between tricuspid valve repair versus replacement. However, valve repair was associated with lower recurrent IE and the need for reoperation. Furthermore, TV repair is associated with a lower need for permanent pacemaker [15]. A multicenter study including 157 patients surgically treated for TVIE from 1983 to 2018 showed that repair of the affected valve does not confer any advantage either at early or long term up to 25 years [32]. In this series, 77 (49%) patients underwent TV repair, 72 (46%) TV replacement, and 8 (5%) prosthetic TV replacement. Overall early mortality was 11% and late mortality was 20%; survival rates at 10, 20, and 25 years were 66%, 60%, and 44%, respectively. Five patients showed IE recurrence on the native or prosthetic TV, and survival free from TVIE recurrence was 65%, 59%, and 43% at 10, 20, and 25 years, respectively. At multivariable analysis, age, prosthetic TV replacement, IVDU, mycotic TVIE, and the presence of cardiac implantable electronic device leads were risk factors for both unfavorable late survival and lower survival free from TVIE recurrence [32]. A recent nationwide cohort study on 704 patients undergoing TV surgery due to IE between 2000 and 2013 showed that 58.5% underwent TV repair and 41.5% underwent TV replacement [33]. After the inverse probability of treatment weighting, the in-hospital mortality rate between the two groups was not significantly different. However, patients who received TV repair had lower rates of perioperative complications, including massive blood transfusion, de novo dialysis, and deep wound infection; longer ICU and hospital stays; and higher hospital cost. Regarding late outcomes, TV repair was associated with lower risks of all-cause readmission, readmission for adverse liver outcomes, new permanent pacemaker implantation, and all-cause mortality than TV replacement.

### Tricuspid valve replacement

Tricuspid valve replacement is still the most commonly performed intervention for TVIE, although consensus guidelines from the American Association for Thoracic Surgery recommend vegetation debridement and repair of the valve (Class I, level of evidence B) [11]. In fact, surgeon and institutional experience for complex TV reconstruction is usually small, and hence, there is a low threshold for replacement, particularly for IE. Additionally, in patients with a total destruction of the TV leaflets, valve replacement may represent the only therapeutic alternative.

Gaca et al. reported the results of 910 operations for TVIE performed between 2002 and 2009 [8] and showed that replacement of the TV was the most common procedure (54%), followed by TV repair (39%) and valvectomy (7%). In this series, 92% of patients undergoing valve replacement received a bioprosthetic valve. Overall operative mortality was 7.3%, with no significant difference in mortality among valvectomy (12%), repair (7.6%), and replacement (6.3%). The majority (69%) of patients in this series had active endocarditis, meaning that they were receiving antibiotic therapy at the time of surgery. Among patients with active endocarditis, the replacement rate was 50%, the repair rate was 40%, and the valvectomy rate was 10%. In comparison, healed patients had a replacement rate of 61%, a repair rate of 37%, and a valvectomy rate of 2%. Compared with the active group, healed patients experienced a trend toward lower operative mortality, lower complication rates, and shorter overall length of stay, suggesting that the conversion of active endocarditis to healed endocarditis could improve the outcomes of surgery for TVIE. Similarly, Slaughter et al. reported that 60% of 1613 patients with intravenous drug-associated TVIE were treated with valve replacement between 2011 and 2016 and found no difference in operative mortality between valve repair and replacement. [20]. Although in this series operative mortality between repair and replacement was similar (2% vs 3%), the authors recommend that, when anatomically possible, repair should be the preferred management for TVIE to potentially avoid recurrent valve infection and prosthetic valve degeneration.

Recently, Xie et al. retrospectively analyzed 56 patients who underwent TV surgery for IE from 2006 to 2019 and found that 59% of the patients underwent TV replacement [34]. The postoperative 30-day mortality rates were similar between TV repair and replacement. However, TV repair was found to be superior to replacement in reducing the need for postoperative blood transfusions, the risk of postoperative complications, and the need for long-term reoperations. The postoperative 3-, 5-, and 7-year survival rates were 100%, 100%, and 95.5% in the repair group and 96.7%, 96.7%, and 96.7% in the replacement group. The 5-year and 10-year reoperation rates were 0% and 0% in the repair group and 6.7% and 20% in the replacement group.

Valve replacement with either a biological or mechanical valve exposes the patient to valve-related complications, heart block requiring a pacemaker, and risk of recurrent endocarditis [15, 16]. When valve replacement is necessary, most authors avoid the use of mechanical prostheses in a tricuspid position [7]. Dawood et al. do not endorse the use of mechanical prosthesis in the tricuspid position [29]. The authors reported excellent durability of tissue valves in the low-pressure right-sided circulation and emphasized their reluctance to implant a thrombogenic mechanical prosthesis with the associated requirement for lifetime warfarin anticoagulation in the TVIE population, in whom IVDU is predominant [29]. Bioprosthetic valves have shown good long-term freedom from structural valve deterioration and the need for reoperation given the low transvalvular gradients in the tricuspid position [35]. In a 25-year study comparing mechanical and bioprosthetic TV replacement in 90 patients, the type of prosthesis was not shown to affect early and late mortality, or reoperation rate [36]. Mortality at 30 days and 5 years respectively was 15% and 27% in the mechanical group, compared to 21% and 30% in the bioprosthetic group, with no significant differences between the two groups. Freedom from reoperation at 5, 10, and 15 years was 86%, 76%, and 70% in the mechanical group, versus 97%, 83%, and 57% in the bioprosthetic group with no significant differences between the two groups [36]. Similarly, a meta-analysis of 1160 prostheses and 6046 follow-up years showed no significant differences in survival and reoperation rates between patients with biological and mechanical prosthetic valve in a tricuspid position [37]. Novel transcatheter approaches have been developed for the treatment of TV regurgitation, also representing a potential modality for re-intervention after failed TV replacement [38]. A multi-center French study has reported successful tricuspid valve-in-valve implantation of Melody (Medtronic Inc., Minneapolis, MN, USA) and SAPIEN (Edwards Lifesciences, Irvine, CA, USA) transcatheter valves in high-risk patients with failing tricuspid bioprostheses [39].

An important concern with TV operations, particularly with valve replacement, is injury to the conduction system. The greater risk of heart block in patients with TV valve replacement may be due to the extension of infection into the region of the cardiac conduction system or as a complication of TV replacement [15]. The overall permanent pacemaker implantation after TV operations has been reported to be 11.6% [40]. In Dawood experience, 7% of the patients required placement of a new postoperative pacemaker before discharge, with rates of 12.5% in the replacement group and 3.1% in the repair group. Placement of a permanent endocardial pacing wiring across a bioprosthesis can result in early failure of the bioprosthesis. Transvalvular pacing wires can result in leaflet perforation, valve damage, and valve dysfunction. A fibrotic response can develop from contact with the lead against leaflet tissue and the subvalvular apparatus. In bioprosthetic valves, this fibrosis can distort the leaflet geometry and result in TR, stenosis, or both. To avoid this, permanent epicardial pacing wires should be placed at the time of the operation for all TV replacements but not for repairs [29].

Homografts have been used in the context of complex infective endocarditis [41] and especially in the case of prosthetic valve endocarditis [42] due to their intrinsic resistance to infection, avoidance of anticoagulation, and superior hemodynamics. Mitral homograft have been used for isolated TVIE [43, 44] and multiple valve IE [45] with good early and late postoperative outcomes. Mestres et al. reported their 6-year experience with the use of mitral homograft in tricuspid position for the treatment of TVIE; the echocardiographic evalutaion showed neither calcification nor rupture of the homograft and different degrees of tricuspid regurgitation with no clinical impact at the end of the follow-up [44]. Furthermore, mitral homograft in the tricuspid position can also be repaired in case of recurrent IE. The authors, in fact, performed a homograft ring annuloplasty 13 years after the original operation with good result [46].

The TV can be also partially replaced with a mitral homograft. Couetil et al. described their technique for partial replacement of the TV with mitral homograft in seven patients with TVIE and complete destruction of a valve leaflet [47]. In three patients, only the anterior leaflet of the mitral homograft was used. The mitral homograft was divided into its two constituent leaflets by dividing the commissural areas of cusp tissue and the papillary muscles between the attachments of anterior and posterior leaflet chordae. In four patients, because of an enlarged tricuspid surface area, the anterior leaflet and the posterior commissure with the adjacent part of the posterior leaflet (P3) of the mitral homograft were used. The homograft is sutured to the tricuspid annulus from left to right, starting at the anteroseptal commissure of the tricuspid valve up to the middle of the anterior mitral leaflet. Then, the anterior papillary muscle of the homograft is inserted in the right ventricle. To determine the correct position of the anterior papillary muscle in the right ventricle, the free margin of the homograft corresponding to the sutured part is pulled against the septal part of the tricuspid annulus in systolic position and temporarily fixed with a stay suture. The anterior papillary muscle is positioned against the free wall of the right ventricle with its cords in tension and in proper orientation. Once the correct position is determined, some trabeculations of the right ventricle are trimmed to create a solid base of implantation. The anterior papillary muscle of the homograft is then sutured with 4–0 polypropylene. The remaining suture of the mitral homograft to the tricuspid annulus is completed up to P3, followed by the implantation of the posterior papillary muscle of the homograft in the right ventricle using the same technique as for positioning the anterior papillary muscle. P3 is sutured side to side to the septal leaflet to expand the surface of this leaflet. An annuloplasty ring is then selected according to the usual criteria and sutured into position.

Pericardial cylinder implantation has been proposed as an alternative in patients with TVIE and the complete destruction of the TV leaflets. This technique allows the replacement of the TV while avoiding the use of prosthetic material [48]. A rectangular patch of 10 × 4 cm autologous pericardium is harvested prior to cardio-pulmonary bypass; alternatively, the cylinder can also be made with a bovine pericardial patch. The shorter sides of the patch are anastomosed with continuous 5–0 polypropylene suture to obtain a cylinder with a final diameter sized to match the native annulus. The length of the cylinder is determined by either the echocardiographic distance from the annulus to the papillary muscle tips or approximately 120% of its diameter. For placement of the cylinder, three points every 120° are marked on the cylinder as the expected attachment points inside the right ventricle. Because the right ventricular papillary muscle number and position may vary, three papillary muscle attachments are chosen, roughly equidistant at 120° apart. The anterior papillary muscle is typically the largest and acts as the reference point for the choice of the other attachments. The posterior papillary muscle is often bifid or trifid, and the septal papillary muscle is variable, and it may be small or multiple or even absent in up to 20% of normal patient, with septal leaflet chordal attachment to the septal papillary muscle or directly to the septal myocardium. After the complete excision of the TV together with all infected tissue, the heads of the papillary muscle are identified, and the previously marked three points of the pericardial cylinder are attached to the tips, base, or body of the selected papillary muscles using 5–0 e-PTFE (Gore-Tex®, L. Gore & Associates, Inc, Flagstaff, Ariz) or polypropylene mattress stitches with or without pledgets. The proximal part of the cylinder is anastomosed to the annulus of the TV using a continuous 5–0 polypropylene suture, gradually compensating the difference in diameter between the native annulus and cylinder [48]. In a series of nine patients with TVIE treated with pericardial cylinder implantation, no recurrence of IE was observed during the follow-up; however, three patients experienced early degeneration with stenosis within 6 months from surgery, requiring balloon valvuloplasty or TV replacement [48]. The concept of the construction of a cylindrical valve made of fully biological material was first tested in the aortic position by Cox et al. [49]. Other series reported the use of a cylinder in the tricuspid position using small intestinal submucosa extracellular matrix (CorMatrix®, CorMatrix Cardiovascular Inc., GA, USA) [50, 51]. In a Canadian study, the largest multicenter study to date, CorMatrix® was used as the material for construction of the cylinder, and polypropylene suture was used for the attachment to the papillary muscles in 18 patients [52]. In this study, four mechanical complications occurred due to ruptures of the attachment of the cylinder to the papillary muscles, or the cylinder itself. In the study by Charkiewicz-Szeremeta, such complications did not occur, maybe because the authors used patients’ own or bovine pericardium to construct the cylinder and 5–0 e-PTFE (Gore-Tex®, L. Gore & Associates, Inc, Flagstaff, Ariz) suture for attachment to the papillary muscles [48]. However, the success of eliminating the recurrence of IE after pericardial cylinder implantation in the tricuspid orifice did not translate into the durability of this valve, because in three cases, rapid degeneration and stenosis of this valve were observed within 6 months after the surgery.

### Percutaneous intracardiac vegetectomy

Several studies have analyzed the efficacy of AngioVac system (AngioDynamics, Latham, NY) for percutaneous intracardiac vegetectomy in patients with TVIE [13, 14]. AngioVac system is a vacuum-based device that was approved in 2014 for the percutaneous removal of undesirable materials from the intravascular system. AngioVac system consists of a venovenous extracorporeal bypass circuit with an external filtration system and a reperfusion cannula to allow blood return. Traditionally, percutaneous vacuum-assisted thrombectomy devices have been primarily used for mechanical thrombectomy in the setting of caval thrombosis and pulmonary emboli [53, 54]. In 2019, a meta-analysis demonstrated that AngioVac and similar thrombectomy systems were safe and effective treatment modalities for right heart vegetations and intracardiac thrombi [55].

Percutaneous vegetectomy is often considered the next best option in the setting of poor surgical candidacy particularly in patients with known IVDU recidivism and/or unstable clinical condition. George et al. reviewed the outcomes of 33 patients with large tricuspid valve vegetations and high surgical risk who underwent percutaneous vegetectomy [56]. All patients survived the procedure with a 61% reduction in size after the procedure, and 90.9% survived the index hospitalization. Three patients proceeded to elective tricuspid valve replacement due to the worsening severity of tricuspid regurgitation [56].

Scantland et al. recently reported on their experience from 2014 with the use of the AngioVac system in 24 patients with TVIE and evaluated the impact of percutaneous vegetectomy on surgical candidacy in these patients [13]. In this series, 30-day mortality was 0%, and technical success was achieved in 90% of the patients. Debulking of intracardiac vegetations ≥ 70% is generally accepted as technically successful by early adopters of percutaneous vegetectomy with values 70–100% considered technically successful according to the Registry of AngioVac Procedure in Detail (RAPID) [57]. Five (20.8%) patients who needed surgical TV repair following percutaneous vegetectomy were for refractory symptomatic TV regurgitation. These findings suggest that percutaneous vegetectomy may improve surgical candidacy and could serve as a bridge if surgery remains indicated after infection source control in patients with previous poor surgical candidacy.

A meta-analysis investigated the efficacy and safety of AngioVac-assisted vegetation debulking in 301 patients with RSIE from 44 studies [14]. The primary outcomes of this study were procedural success and clinical success. The procedural success was defined as the ability of AngioVac to produce residual vegetation size < 50% without serious procedural complications (including access site complications requiring intervention, pericardial effusion or tamponade, dissection, perforation, embolic phenomena, or intraoperative mortality). The clinical success was defined as a composite definition of residual vegetation size < 50%, in-hospital survival, absence of persistent bacteremia, and valve function not requiring further intervention. This metanalysis showed procedural success and clinical success of 89.2% and 79.1%, respectively. Vegetation removal > 50% was achieved in 90%, and bacteremia clearance was achieved in 82.5% of patients, while the procedure-related complications were noted in 10.1% of patients. These results confirm that the AngioVac device has an acceptable efficacy and safety profile in extracting RSIE vegetations, making it a viable alternative therapeutic option for poor surgical candidates.

## Conclusion

Tricuspid valve infective endocarditis is an uncommon disease that can be mainly treated with medical treatment. When surgery is indicated, the appropriate surgical option still remains to be determined, and the ultimate decision regarding the surgical approach in patients with TVIE is made intraoperatively. Repair is considered most desirable, leaving the patient with a native functional valve and no prosthetic material. This is generally achieved through extensive debridement of the leaflet vegetation and primary repair. In situations where the leaflet could be primarily patch repaired, the annulus should be reconstructed and supported with a suture or prosthetic ring annuloplasty. However, given the destructive nature of the bacteria encountered in these patients, this is not always possible. Often, the choice is one between replacement of the valve and complete valvectomy. Persistent sepsis, abscess formation, ongoing drug use, and refusal of rehabilitation are all factors in deciding which patients would undergo valvectomy. Tricuspid valvectomy may also serve as a bridge to following valve replacement. In patients with unstable clinical conditions and a high risk of recidivism, percutaneous vegetectomy may represent an alternative to increase patients’ surgical candidacy.

### Supplementary Information

Below is the link to the electronic supplementary material.

Supplementary file1 (MOV 2667 KB)

Supplementary file2 (MOV 1995 KB)

## Data Availability

Not applicable.
